# Risk factors outperform intracranial large artery stenosis predicting unfavorable outcomes in patients with stroke

**DOI:** 10.1186/s12883-019-1408-1

**Published:** 2019-08-01

**Authors:** K. C. Chang, I. C. Chuang, Y. C. Huang, C. Y. Wu, W. C. Lin, Y. L. Kuo, T. H. Lee, S. J. Ryu

**Affiliations:** 1 Division of Cerebrovascular Diseases, Department of Neurology, Chang Gung Memorial Hospital, Linkou, Taiwan; 2 Discharge Planning Service Center, Chang Gung Memorial Hospital, Kaohsiung, Taiwan; 3 College of Medicine, Chang Gung University, Taoyuan, Taiwan; 4 Department of Occupational Therapy and Graduate Institute of Behavioral Sciences, College of Medicine, Chang Gung University, Taoyuan, Taiwan; 5 Department of Measurement and Statistics, Education, National University of Tainan, Tainan, Taiwan; 6 Department of Physical Medicine and Rehabilitation, Healthy Aging Research Center at Chang Gung University, Chang Gung Memorial Hospital at Linkou, 259 Wen-hwa 1st Road, Taoyuan, Taiwan; 7 Department of Radiology, Chang Gung Memorial Hospital, Kaohsiung, Taiwan

**Keywords:** Intracranial arterial stenosis, Infarction, Prognosis, MRI

## Abstract

**Background:**

This study examined how intracranial large artery stenosis (ILAS), symptomatic and asymptomatic ILAS, and risk factors affect unfavorable outcome events after medical treatment in routine clinical practice.

**Methods:**

This was a 24-month prospective observational study of consecutively recruited stroke patients. All participants underwent magnetic resonance angiography, and their clinical characteristics were assessed. Outcome events were vascular outcome, recurrent stroke, and death. Cox regression analyses were performed to identify potential factors associated with an unfavorable outcome, which included demographic and clinical characteristics, the risk factors, and stenosis status.

**Results:**

The analysis included 686 patients; among them, 371 were assessed as ILAS negative, 231 as symptomatic ILAS, and 84 as asymptomatic ILAS. Body mass index (*p* < .05), hypertension (*p* = .01), and old infarction (*p* = .047) were factors relating to vascular outcomes. Hypertension was the only factor for recurrent stroke (*p* = .035). Poor glomerular filtration rate (< 30 mL/min/1.73 m^2^) (*p* = .011) and baseline National Institutes of Health Stroke Scale scores (*p* < .001) were significant predictors of death.

**Conclusions:**

This study extended previous results from clinical trials to a community-based cohort study by concurrently looking at the presence/absence of stenosis and a symptomatic/asymptomatic stenotic artery. Substantiated risk factors rather than the stenosis status were predominant determinants of adverse outcome. Although the degree of stenosis is often an indicator for treatment, we suggest risk factors, such as hypertension and renal dysfunction, should be monitored and intensively treated.

## Introduction

Intracranial large artery stenosis (ILAS) is a leading cause of ischemic stroke, especially for Asians and Africans [1, 2]. Several large clinical trials [3–9] have focused on treating intracranial stenosis in which stenosis severity was used to guide treatment decisions. However, most of these trials failed to demonstrate the superior benefits of these treatments to reduce recurrent stroke in patients with ILAS, and the stenting treatment was not better than aggressive medical treatment for preventing recurrent stroke [10]. In addition, the WASID trial and other clinical findings pointed out that risk factors play important roles in reducing the recurrence of stroke or vascular events [5, 11–13]. Given these results, the role of stenosis for unfavorable outcomes is still uncertain. The need to re-identify factors for these outcomes in community-dwelling patients with ILAS is warranted.

These aforementioned studies were limited by patient selection bias (patients coming from clinical trials), no comparable control groups (no recruitment of patients without stenosis), and only consideration of stenosis without inclusion of possible risk factors [13]. Only one study recruited community-based participants to investigate the characteristics and outcomes of patients with vs without ILAS [2]. Controversial evidence for the need to treat asymptomatic ILAS to reduce the risk of an unfavorable outcome was recently reported [14, 15]. The significance of asymptomatic ILAS awaits further investigation.

The primary aim of this study was to examine how the status of ILAS and risk factors in patients with ischemic stroke affect unfavorable outcomes after medical treatment in routine clinical practice. ILAS status was categorized as patients with ILAS vs without ILAS. Patients with ILAS were further divided into the subgroups of patients with asymptomatic vs symptomatic ILAS.

## Methods

### Study design and participants

This was a 24-month prospective observational single hospital-based study that consecutively recruited ischemic stroke patients. The study enrolled patients who met all of the following eligibility criteria: (1) patients with acute ischemic stroke or transient ischemic attack; (2) patients without intracerebral hemorrhage; (3) patients who had received magnetic resonance (MR) imaging (MRI)/MR angiography (MRA) screening in the acute hospitalization; and (4) patients who provided written informed consent. The institutional review board of the Chang Gung Memorial Hospital at Kaohsiung Medical Center approved the study protocols [16].

### Assessment of patient characteristics

Demographic and clinical characteristics were assessed. The risk factors included body mass index (BMI), internal carotid artery (ICA) stenosis, glomerular filtration rate (GFR), serum uric acid level, baseline neurologic deficits, and history of ischemic stroke, hypertension, diabetes mellitus (DM), hyperlipidemia, heart disease (congestive heart failure, valvular heart disease, and history of cardiac disease), atrial fibrillation, and smoking [17]. Clearer exclusion criteria or classification criteria and corresponding analysis may eliminate those biases. A BMI of ≥27 kg/m^2^ was categorized as indicating obesity and 24 to 27 kg/m^2^ as overweight. ICA stenosis was classified as > 50% and ≤ 50%. ICA was evaluated by carotid duplex imaging during the acute hospitalization, with stenosis > 50% defined as a peak systolic velocity ≥ 140 cm/s or as a ratio of the ICA peak systolic velocity to the common carotid artery peak systolic velocity of > 2 [18]. A neurologist evaluated the ICA ratings. Three investigators discussed and decided the ratings together when the quality of the carotid duplex images was suboptimal. The creatinine clearance rate was estimated using the Cockcroft-Gault formula. GFR < 60 mL/min/1.73 m^2^ was assessed as abnormal, and results were further categorized into two groups with above or below 30 mL/min/1.73 m^2^. A serum uric acid level of > 8.3 mg/dL was assessed as abnormal.

Baseline neurologic deficits at admission were assessed with National Institutes of Health Stroke Scale (NIHSS), with a scale from 0 to 38 (0 = normal). In addition, the stroke etiology (the subtype of recurrent stroke) might have a large effect on the outcome of recurrent stroke; thus, the subtype of recurrent stroke was further classified into small vessels occlusive, cardioembolism, and atherosclerosis.

### Image analysis/participant classification

All participants underwent MRA on a 1.5-T MR scanner (the Philips Gyroscan Intera or the GE Signa). Although MRA 3 T is available and outperformed MRA 1.5 T in various outcomes, such as improved signal-to-noise ratio and higher resolution, its use is usually limited to the research setting. However, MRA 1.5 T is routinely used in clinics and is easily accessible to scan a large number of patients. Three-dimension time of flight was used. The image was usually reconstructed from 80 images insonated centered at the sella and 19 images from the sagittal plane. Brain MRA was used to assess 13 segments of intracranial large arteries: bilateral intracranial ICA, first and second part of the middle cerebral arteries, anterior cerebral arteries, posterior cerebral arteries, vertebral arteries, and basilar artery. Because the criteria used for stenosis evaluation on a cerebral angiogram are not easily adapted for MRA, a modified method to clarify ILAS was explored [3, 19, 20]. In this study, source and maximum intensity projection images were both available for MRA. The single view with the highest percentage of stenosis was measured. Four-scale grading of ILAS severity was developed by the study team: (1) mild: no obliteration of flow of a vessel, (2) moderate: obliteration of flow between two segments of a vessel, (3) severe: flow void between two segments of a vessel, and, (4) occlusion: no flow after a specific point of a vessel.

For descriptive purpose, we categorized patients as patients without ILAS (ILAS–), including the mild group, and patients with ILAS (ILAS+), including moderate, severe, and occlusion groups. ILAS+ was further categorized into two groups: symptomatic ILAS+ (ILAS+S), defined as ILAS with corresponding acute infarct detected by an MRI diffusion-weighted image or clinical symptoms, regardless the presence or absence of asymptomatic ILAS+ (ILAS+AS), and ILAS+AS as ILAS coexisting with no corresponding infarction. The number of ILAS+ was classified as single if only one stenotic segment was recognized and as multiple if more than one stenotic segment was found.

The ILAS in this study was documented by board-certified neuroradiologists unaware of the patients’ recruitment status. After recruitment was completed, one neurologist blinded to the outcomes of patients confirmed the stenosis ratings. If no focal stenosis was identified, although smaller in caliber compared with the other side, the vessel would be assessed as no ILAS [20, 21]. If the quality of images from MRI/MRA was suboptimal, a panel of three investigators decided the grading. Old infarction identified by brain MRI was reported by neuroradiologists and retrospectively confirmed by one investigator without information on the clinical presentation.

### Assessment of outcome events

Because of the universal health insurance program in the study area with a single payer and limited copayment, MRI use was not confounded by socioeconomic status [21, 22]. Approximately 70% of admitted patients with ischemic stroke in this study hospital would have undergone brain MRI, including MRA, in the acute hospitalization. After acute hospitalization, patients usually visited clinics every 1 to 3 months for prescriptions to control risk factors and for stroke prevention. Therapies to prevent vascular events were similar to prophylactic medical or surgical therapies used for high-risk patients. Concomitant medication data were not collected in detail, but the inequality of care related to the incidence of outcome events was carefully checked. To address efficacy of care, we categorized patients as monitored in the study hospital or other care facilities and categorized risk factors as regularly treated, irregularly treated, or not treated. The scheduled visits of this observational study were every 6 months after enrollment by investigators. All patients were to be monitored until death or the end of the study.

The outcome events were (1) vascular outcome events, defined as cerebrovascular with recurrent stroke; cardiovascular event as any cardiovascular diseases requiring revascularization or admission; renovascular event as serum creatinine level increased by two times during follow-up, newly initiated hemodialysis, or diagnosis of nephropathy; or other vascular disease with vascular entity-related diagnosis at any new admission or emergency department visits, at the discretion of the investigators; (2) recurrent stroke, defined as an ischemic or hemorrhagic stroke after the first index stroke during the follow-up period; and (3) death. Outcome events were verified by hospital records and ascertained by one neurologist.

### Statistical analysis

SPSS 16.0 software (SPSS Inc., Chicago, IL, USA) was used for data analyses. The χ^2^ test was used to determine significant differences in attributes studied in relation to ILAS. Cox regression was used to assess how ILAS+ symptomatic/asymptomatic and risk factors affected unfavorable outcome events. The variables included in the Cox regression analysis were sex, BMI, ICA stenosis, GFR, serum uric acid level, baseline neurologic deficits, and history of ischemic stroke, hypertension, DM, hyperlipidemia, heart disease, atrial fibrillation, smoking, multiple stenosis, and stenosis status. In the Cox regression, variables of interest were backward selected in the models. We further performed univariate analysis using the etiologic classification as the potential predictor of recurrent stroke and also multivariate Cox regression analysis involving the other variables together with the variable of stroke etiology as the potential predictors. All significant tests were two-tailed, and differences were considered to be statistically significant at a *p* < .05 level.

## Results

**Fig. 1:**
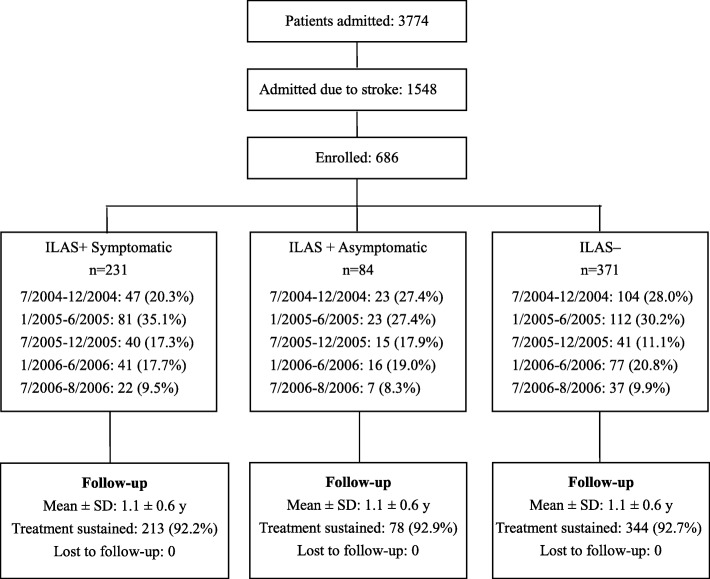
Recruitment and follow-up flowchart

The study recruited 686 patients [Fig. 1]; among these, 315 (45.9%) were assessed as ILAS+ and 371 (54.1%) as ILAS−, and among the ILAS+ patients, 231 (33.7%) were assessed as ILAS+S and 84 (12.2%) as ILAS+AS. Vessel occlusions were documented in 63 patients. The brain MRI was done 4.5 ± 5.6 days after stroke onset. Throughout the study, modifiable risk factors were regularly treated in 213 ILAS+S (92.2%), 78 ILAS+AS (92.9%), and 344 ILAS− (92.7%) patients. All participants were monitored for 1.1 (standard deviation, 0.6) years on average. During this period, 91 participants had a vascular outcome, 57 participants had a recurrent stroke, and 51 participants died.

The baseline characteristics of the participants in the ILAS+S, ILAS+AS, and ILAS− groups are summarized in Table 1. There were significant differences among the three groups in the incidences of extracranial carotid artery stenosis > 50% and baseline NIHSS scores. History of previous stroke was documented by medical history in 34.5 to 41.6% of patients, and an old infarction was evident by brain MRI in 66.7 to 79.8% of patients. No significant differences were found among the three groups on treatment frequency of risk factors, indicating that control of risk factors during the study period might not be a confounder.

**Table 1:** Characteristics of 686 patients enrolled

	Total (*N* = 686)	ILAS+S (*n* = 231, 33.7%)		ILAS+AS (*n* = 84, 12.2%)		ILAS-(*n* = 371, 54.1%)		*P*
		N	(%)	N	(%)	N	(%)	
Age, y								
< 65	265	82	(35.5)	42	(50)	141	(38)	0.061
≥ 65	421	149	(64.5)	42	(50)	230	(62)	
Sex								
Female	250	88	(38.1)	34	(40.5)	128	(34.5)	0.48
Male	436	143	(61.9)	50	(59.5)	243	(65.5)	
Body mass index, kg/m^2^								0.624
< 24	306	101	(43.7)	44	(52.4)	161	(43.4)	0.514
24–27	230	81	(35.1)	24	(28.6)	125	(33.7)	
≥ 27	150	49	(21.2)	16	(19.)	85	(22.9)	
Carotid artery stenosis								
< 50%	602	180	(85.7)	72	(91.1)	350	(96.7)	< 0.001
≥ 50%	49	30	(14.3)	7	(8.9)	12	(3.3)	
Glomerular filtration rate								
≥ 60	345	103	(44.6)	45	(53.6)	197	(53.2)	0.084
30–59	285	113	(48.9)	33	(39.3)	139	(37.6)	
< 30	55	15	(6.5)	6	(7.1)	34	(9.2)	
Uric acid		5.9	±4.4	5.9	±1.9	5.6	±1.8	0.36
Normal	597	190	(86.4)	72	(86.7)	335	(91.5)	0.109
Abnormal	72	30	(13.6)	11	(13.3)	31	(8.5)	
History of ischemic stroke	254	96	(41.6)	30	(35.7)	128	(34.5)	0.211
Hypertension	481	170	(73.6)	66	(78.6)	245	(66)	0.028
Diabetes mellitus	277	94	(40.7)	42	(50)	141	(38)	0.128
Hyperlipidemia	386	144	(62.3)	47	(56)	195	(52.6)	0.063
Heart disease	171	62	(26.8)	21	(25)	88	(23.7)	0.69
Atrial fibrillation	50	17	(7.4)	8	(9.5)	25	(6.7)	0.674
Smoking	238	83	(35.9)	24	(28.6)	131	(335.3)	0.448
Baseline NIHSS								
0–6	413	120	(51.9)	55	(65.5)	238	(64.2)	< 0.001
7–15	198	66	(28.6)	20	(23.8)	112	(30.2)	
16–38	75	45	(19.5)	9	(10.7)	21	(5.7)	
Old infarction	480	154	(66.7)	67	(79.8)	259	(69.8)	0.081
Multiple ILAS	185	144	(62.3)	41	(48.8)	0	(0)	
Risk factors control treatment								
Regular treated	635	213	(92.2)	78	(92.9)	344	(92.7)	0.997
Not treated	40	14	(6.1)	5	(6.0)	21	(5.7)	
Irregular	11	4	(1.7)	1	(1.2)	6	(1.6)	
Follow								
Follow-up in the study hospital	587	189	(86.5)	77	(91.7)	321	(81.8)	0.066
Follow-in other care facilities	99	42	(13.5)	7	(8.3)	50	(18.2)	

§ *ILAS* Intracranial Large Artery Stenosis, *ILAS+S* symptomatic ILAS, *ILAS+AS* asymptomatic ILAS, *NIHSS* National Institutes of Health Stroke Scale

Tables 2 and 3 report hazard ratios (HRs) with corresponding 95% confidence intervals (CIs) for outcome events by characteristics of the patients. Significant factors in the univariate analysis associated with the vascular outcome were GFR, hypertension, heart disease, and old infarction. The multivariate analysis demonstrated a BMI of 24 to 27 kg/m^2^ (HR, 0.59; 95% CI, 0.35 to 0.97; *p* = .039) and > 27 kg/m^2^ (HR, 0.55; 95% CI, 0.30 to 1.00; *p* = .049), hypertension (HR, 2.18; 95% CI, 1.20 to 3.94; *p* = .01), and old infarction (HR, 1.74; 95% CI, 1. 01 to 2.99; *p* = .047) were factors relating to the vascular outcome [Table 3]. However, the univariate and multivariate analysis showed hypertension was the only factor (HR, 2.20; 95% CI, 1.06 to 4.58; *p* = .035) accounting for the recurrent stroke. For death, the univariate analysis showed sex, GFR, uric acid, DM, heart disease, atrial fibrillation, and baseline neurologic deficits were significant factors, and the multivariate analysis showed poor GFR (< 30 mL/min/1.73 m^2^; HR, 3.79; 95% CI, 1.36 to 10.6; *p* = .011) and poor baseline NIHSS scores (> 16; HR, 9.98; 95% CI, 4.35 to 22.91; *p* < .001) were significant.

**Table 2:** Predictors of vascular outcome, recurrent stroke, and death in 686 patients (univariate analysis)

	Vascular outcome			Recurrent stroke			Death		
	Hazard Ratio (95%CI)		*P* Value	Hazard Ratio (95%CI)		*P* Value	Hazard Ratio (95%CI)		*P* Value
Age, y									
≤ 64	1			1			1		
≥ 65	1.169	(.762–1.792)	.474	1.140	(.666–1.953)	.633	1.449	(.802–2.618)	.219
Sex									
Female	1			1			1		
Male	1.026	(.667–1.578)	.907	1.218	(.697–2.129)	.490	.526	(.304–.912)	.022
Body mass index, kg/m^2^									
< 24	1			1			1		
24–27	.740	(.464–1.179)	.205	.820	(.453–1.482)	.511	.800	(.438–1.461)	.467
≥ 27	.600	(.336–1.071)	.084	.762	(.379–1.530)	.444	.418	(.173–1.008)	.052
Carotid artery stenosis									
≤ 50%	1			1			1		
> 50%	1.072	(.495–2.320)	0.860	1.488	(.638–3.470)	0.358	0.044	(0–6.445)	.220
Glomerular filtration rate									
≥ 60	1			1			1		
30–59	1.305	(.840–2.029)	.237	1.171	(.683–2.008)	0.567	1.956	(1.039–3.684)	.038
< 30	2.181	(1.117–4.258)	.022	1.048	(.367–2.992)	0.931	4.958	(2.298–10.695)	<.001
Uric acid									
Normal	1			1			1		
Abnormal	1.583	(.879–2.851)	.126	1.495	(.707–3.160)	.293	2.269	(1.132–4.545)	.021
Hypertension	2.094	(1.203–3.642)	.009	2.187	(1.073–4.457)	.031	1.178	(.628–2.212)	.610
Diabetes mellitus	1.375	(.912–2.075)	.129	1.231	(.731–2.073)	.435	1.753	(1.010–3.044	.046
Hyperlipidemia	1.023	(.675–1.550)	.914	1.141	(.672–1.937)	.625	.861	(.497–1.492)	.594
Heart disease	1.678	(1.095–2.574)	.018	1.143	(.641–2.037)	.651	2.226	(1.279–3.875)	.005
Atrial fibrillation	1.466	(.709–3.033)	.302	.512	(.125–2.102)	.353	2.345	(1.055–5.213)	.036
Smoking	.923	(.600–1.420)	.715	.942	(.547–1.625)	.831	.540	(.282–1.031)	.062
Baseline NIHSS									
0–6	1			1			1		
7–15	.766	(.411–1.428)	.401	.754	(.395–1.440)	.392	2.709	(1.306–5.620)	.007
16–38	.765	(.379–1.547)	.456	.774	(.305–1.961)	.588	10.003	(5.116–19.561)	<.001
Old infarction	1.804	(1.087–2.992)	.022	1.572	(.846–2.921)	.152	1.843	(.923–3.680)	.083
Stenosis state									
ILAS-	1			1			1		
ILAS+AS	1.657	(.951–2.886)	.075	1.370	(.622–3.018)	.435	2.115	(.996–4.963)	.051
ILAS+S	.977	(.608–1.568)	.922	1.384	(.788–2.431)	.258	1.585	(.895–2.924)	.141
Multiple stenosis	1.213	(.773–1.901)	.401	1.224	(.694–2.158)	.486	1.555	(.875–2.761)	.132
Subtypes of stroke									
Small vessel occlusive	–			1		.595	–		
Atherosclerosis	–	–	–	.483	(.035–6.636)	.587	–	–	–
Cardioembolism	–	–	–	.909	(.075–11.027)	.941	–	–	–

§ *ILAS* Intracranial Large Artery Stenosis, *ILAS+S* symptomatic ILAS, *ILAS+AS* asymptomatic ILAS, *NIHSS* National Institutes of Health Stroke Scale

**Table 3:** Multivariate Cox proportional-hazard regression analysis for vascular outcome, recurrent stroke, and death (*N* = 685)

	Vascular outcome			Recurrent stroke			Death		
	Hazard Ratio (95%CI)		*P* Value	Hazard Ratio (95%CI)		*P* Value	Hazard Ratio (95%CI)		*P* Value
Age, y									
≤ 64	–			–		–	–		
≥ 65	–			–		–	–		
Sex									
Female	1			–		–	1		
Male	1.146	(.685–1.918)	.605	–		–	.681	.311–1.492	.337
Body mass index, kg/m^2^									
< 24	1			–		–	–		
24–27	.587	(.354–.974)	.039	–		–	–		
≥ 27	.550	(.303–.997)	.049	–		–	–		
Carotid artery stenosis									
≤ 50%	–			–		–	–		
> 50%	–			–		–	–		
Glomerular filtration rate									
≥ 60	–			–		–	1		
30–59	–			–		–	1.965	.875–4.415	.102
< 30	–			–		–	3.793	1.357–10.596	.011
Uric acid									
Normal	1			–		–	1		
Abnormal	1.697	(.881–3.271)	.114	–		–	2.021	.842–4.854	.115
Hypertension	2.176	(1.202–3.939)	.010	2.200	(1.058–4.576)	.035	–		
Diabetes mellitus	–			–		–	–		
Hyperlipidemia	–			–		–	–		
Heart disease	–			–		–	–		
Atrial fibrillation	–			–		–	–		
Smoking	.773	(.460–1.300)	.332	.701	(.374–1.315)	.269	–		
Baseline NIHSS									
0–6	1			–		–	1		
7–15	.936	(.551–1.589)	.806	–		–	2.011	.898–4.504	.089
16–38	1.438	(.711–2.908)	.312	–		–	9.978	4.345–22.914	<.001
Old infarction	1.735	(1.008–2.987)	.047	–		–	1.393	.560–3.465	.476
Stenosis state									
ILAS-	1			–		–	1		
ILAS+AS	1.180	(.650–2.139)	.587	–		–	1.644	.688–4.048	.280
ILAS+S	.842	(.506–1.402)	.508	–		–	.886	.408–1.927	.760
Multiple stenosis	–			–		–	–		
Subtypes of stroke									
Small vessel occlusive	–			–		–	–		
Atherosclerosis	–	–	–	–		–	–	–	–
Cardioembolism	–	–	–	–		–	–	–	–

§ *ILAS* Intracranial Large Artery Stenosis, *ILAS+S* symptomatic ILAS, *ILAS+AS* asymptomatic ILAS, *NIHSS* National Institutes of Health Stroke Scale

## Discussion

To date, the optimal treatment of ILAS has not been confirmed [23]. The current study attempted to identify the role of stenosis, in addition to risk factors, for unfavorable outcomes in an effort to augment treatment efficacy. This study with stroke patients outside the clinical trial settings demonstrated that the risk factors, but not the status of stenosis, were the most significant predictors for unfavorable outcome events after medical treatment rather than the stenosis status. The current data suggested that aggressively controlling risk factors might be a prerequisite for preventing adverse outcome events in patients with stroke.

The results of this study indicate that risk factors were associated with worsening disease activity. Consistent with previous studies [3, 12, 24–28], our study provided data supporting the need to control risk factors in patients with ILAS. The findings showed that hypertension was one of the factors relating to vascular outcome events and the independent factor to recurrent stroke. In general, hypertension may stiffen arteries and progress artery disease, possibly relating to an increased risk of vascular events and recurrent stroke. Old infarction and BMI, along with hypertension, were related to vascular outcome events. Overweight (BMI, 24–27 kg/m^2^) and obese (BMI ≥ 27 kg/m^2^) patients have a lower incidence of vascular outcome events than patients with a BMI of < 24 kg/m^2^. Orpana et al. [29] suggested that being overweight (BMI, 25–29.9 kg/m^2^) has a significant protective effect for all-cause mortality compared with being underweight (BMI, 18.5 kg/m^2^). A scrutiny of our data showed that half of our obese patients had a BMI of 27 to 30 kg/m^2^. The protective nature associated with a low incidence of undesirable outcomes may have merit in the overweight and obese patients in this study.

GFR, uric acid, and baseline neurologic deficits (NIHSS admission score) were factors relating to death outcomes in our study. Chronic kidney disease may trigger vascular damage and endothelial dysfunction [30], and previous studies indentified renal dysfunction or chronic kidney disease as strong risk factors of stroke [26, 27, 30]. This study further suggested that renal dysfunction or chronic kidney disease in patients with cerebrovascular disease may be relevant to death. Patients with severe neurologic impairments, as represented by an NIHSS score > 15, have higher incidence of an adverse outcome than those with mild impairments; therefore, the NIHSS score could provide prognostic information to patients and clinicians.

Concurrently considering other relevant characteristics relating to the outcome events, neither ILAS+ and ILAS– nor ILAS+S and ILAS+AS were significant predictors for unfavorable outcome events. Thus, the presence or absence of stenosis and the presence of asymptomatic or symptomatic stenosis were not prominent factors for the outcome events. Although our finding is counterintuitive with the long-standing assumption that stenosis severity is an indicator for treatment decisions to reduce adverse outcome, it reinforces observations from the recent clinical trial that the degree of stenosis does not predict an adverse outcome after treatment with intracranial stents [10]. Our results extend the results from clinical trials with only patients with symptomatic stenosis to a community-based cohort study with patients with symptomatic and asymptomatic stenosis. The reason for stenosis being a non-significant predictor might be that the pathophysiologic mechanisms of recurrent stroke or adverse events in patients with intracranial stenosis are attenuated over time [6, 31–33].

However, our finding is inconsistent with the previous cohort study [3] that occlusion was a significant predictor. The differences between these two studies are in the criterion for recurrent stroke and the classification of stenosis. The previous study included recurrent stroke, targeting only at the territory of the stenotic artery, whereas the present study included all types of recurrent stroke. Furthermore, the present study did not dichotomize lesions with stenosis vs occlusion or near occlusion, whereas the previous study did. We argued that the differentiation between these two is arbitrary and that the accuracy might be questionable [2]. In light of these results, an alternative to lesion stratification according to stenosis might be considered. For example, hypoperfusion vs non-hypoperfusion symptoms [11] or intracranial plaque vs plaque-negative stenosis [13] might deserve further investigation for their possible roles in relating to adverse outcome.

That the participants in this study did not come from clinical trials is noteworthy. They were not population-based but from a metropolitan medical center. However, the incidence of carotid artery stenosis identified by duplex as > 50% was 3 to 14% and higher in patients with ILAS (ILAS+S and ILAS+AS), which was compatible with the incidence reported in the previous study using population-based data [18], indicating the data obtained in the present study should be representative of the general stroke population.

The incidence of patients with ILAS+S and ILAS+AS in this study was similar to the previous study [2], which might be as expected in the Asian population, where the burden of stroke is tremendous. Brain MRI identified an old infarction in > 60% of the study patients, which is higher than the incidence of medical history of ischemic stroke of 33.8 to 41.2%. It might be that patients the present study underwent vascular screening using MRI, whereas the history of previous stroke was documented by computed tomography screening. MRI is more sensitive than computed tomography for detecting ischemic injury, which made the high incidence of old infarction found in the present study acceptable. To avoid overestimation of ILAS by MRA, the visual-based grading of severity of ILAS in this study was modified from the previous design [3, 19] and might be compatible with the methods used in acute stroke trials [6].

Our study has limitations. First, our study was subject to bias because participants were recruited in one medical center. However, the study hospital is the main referral hospital for all types of stroke in the Kaohsiung metropolitan area of Taiwan. The incidence of carotid artery stenosis in the present study was compatible with the incidence reported in the previous study using population-based data, suggesting the data are representative of the population scenario.

Second, owing to limited research manpower, detailed information on concomitant medication or modification of risk factor control is lacking, which might have introduced errors in the analysis of the incidence of outcome events. However, we attempted to reduce such bias in terms of reporting that patients had high rates of being monitored in the study hospital and of being treated for risk factors.

Third, there is no universally accepted standard to diagnose ILAS. In this study, we used MRA and explored a modified method to clarify the stenosis of ILAS based on the study by Chimowitz et al. [3], which should be arguably acceptable.

Fourth, there are several imaging modalities, such as MRA, computed tomographic angiography (CTA), and digital subtraction angiography (DSA), can be used to assess intracranial stenosis, we used MRA to identify ILAS instead of DSA and CTA, which are more sensitive. DSA is considered the gold standard for diagnosing intracranial vascular diseases, and CTA may provide higher diagnostic accuracy of the intracranial atherosclerosis than MRA [34]. DSA, however, can cause some complications, such as transient neurologic deficits [34]. CTA may expose patients to high radiation doses, and the probability of cancer and other biological effects is increased [35]. MRA does not emit damaging ionizing radiation, so that patients are not exposed to these harmful effects. Owing to practical considerations and feasibility, we chose MRA to identify ILAS.

Fifth, some factors that might influence the incidence of outcomes, such as lesion stability, were not included. We recommend that future studies consider these factors.

## Conclusions

This study extended previous results from clinical trials to a community-based cohort study concurrently investigating the presence/absence of stenosis, symptomatic/asymptomatic stenotic artery, and substantial risk factors. Hypertension, BMI, and renal dysfunction status are among the substantial risk factors that are predominant determinants of an adverse outcome rather than the stenosis. We suggest that although the degree of stenosis is often an indicator for treatment, risk factors should be monitored and intensively treated. Future research might consider other classification systems, such as perfusion symptoms or features of the plaque, instead of the type of stenosis, as potential factors of predicting adverse outcome.

## Data Availability

All relevant data are presented in the manuscript. The data sets generated and analyzed during this study are available after the approval of the corresponding authors on reasonable request.

## Abbreviations

BMI: Body mass index
CIs: Confidence intervals
DM: Diabetes mellitus
GFR: Glomerular filtration rate
HRs: Hazard ratios
ICA: Internal carotid artery
ILAS: Intracranial large artery stenosis
ILAS+AS: Asymptomatic ILAS+
ILAS+S: Symptomatic ILAS+
MRA: Magnetic resonance angiography
MRI: Magnetic resonance imaging
NIHSS: National Institutes of Health Stroke Scale

## References

[1] Gorelick PB, Wong KS, Bae HJ, Pandey DK (2008). Large artery intracranial occlusive disease: a large worldwide burden but a relatively neglected frontier. Stroke..

[2] Wang Y, Zhao X, Liu L, Soo YO, Pu Y, Pan Y, Wang Y, Zou X, Leung TW, Cai Y, Bai Q, Wu Y, Wang C, Pan X, Luo B, Wong KS, CICAS Study Group (2014). Prevalence and outcomes of symptomatic intracranial large artery Stenoses and occlusions in China: the Chinese intracranial atherosclerosis (CICAS) study. Stroke..

[3] Chimowitz, Kokkinos J, Strong J, Brown MB, Levine SR, Silliman S, Pessin MS, Weichel E, Sila CA, Furlan AJ, Kargman DE, Sacco RL, Wityk RJ, Ford G, Fayad PB (1995). Warfarin-aspirin symptomatic intracranial disease study group. The warfarin-aspirin symptomatic intracranial disease study. Neurology..

[4] Chimowitz MI, Lynn MJ, Derdeyn CP, Turan TN, Fiorella D, Lane BF, Janis LS, Lutsep HL, Barnwell SL, Waters MF, Hoh BL, Hourihane JM, Levy EI, Alexandrov AV, Harrigan MR, Chiu D, Klucznik RP, Clark JM, McDougall CG, Johnson MD, Pride GL, Torbey MT, Zaidat OO, Rumboldt Z, Cloft HJ, SAMMPRIS trial investigators (2011). Stenting versus aggressive medical therapy for intracranial arterial stenosis. N Engl J Med.

[5] Derdeyn CP, Chimowitz MI, Lynn MJ, Fiorella D, Turan TN, Janis LS, Montgomery J, Nizam A, Lane BF, Lutsep HL, Barnwell SL, Waters MF, Hoh BL, Hourihane JM, Levy EI, Alexandrov AV, Harrigan MR, Chiu D, Klucznik RP, Clark JM, CG MD, Johnson MD, Pride GL, Lynch JR, Zaidat OO, Rumboldt Z, Cloft HJ, Stenting and Aggressive Medical Management for Preventing Recurrent Stroke in Intracranial Stenosis Trial Investigators (2014). Aggressive medical treatment with or without stenting in high-risk patients with intracranial artery stenosis (SAMMPRIS). The final results of a randomised trial. Lancet.

[6] Albers GW, von Kummer R, Truelsen T, Jensen JK, Ravn GM, Grønning BA, Chabriat H, Chang KC, Davalos AE, Ford GA, Grotta J, Kaste M, Schwamm LH, Shuaib A, DIAS-3 investigators (2015). Safety and efficacy of desmoteplase given 3-9 h after ischaemic stroke in patients with occlusion or high-grade stenosis in major cerebral arteries (DIAS-3). A double-blind, randomised, placebo-controlled phase 3 trial. Lancet Neurol.

[7] Jiang W-J, Du B, Leung TW, Xu XT, Jin M, Dong KH (2007). Symptomatic intracranial stenosis: cerebrovascular complications from elective stent placement. Radiology..

[8] Samaniego EA, Hetzel S, Thirunarayanan S, Aagaard-Kienitz B, Turk AS, Levine R (2009). Outcome of symptomatic intracranial atherosclerotic disease. Stroke..

[9] Zaidat OO, Klucznik R, Alexander MJ, Chaloupka J, Lutsep H, Barnwell S, Mawad M, Lane B, Lynn MJ, Chimowitz M, NIH multicenter wingspan, intracranial stent, registry study, group (2008). The NIH registry on use of the wingspan stent for symptomatic 70-99% intracranial arterial stenosis. Neurology..

[10] Alexander MD, Cooke DL, Meyers PM, Amans MR, Dowd CF, Halbach VV (2016). Lesion stability characteristics outperform degree of stenosis in predicting outcomes following stenting for symptomatic intracranial atherosclerosis. J Neurointerv Surg.

[11] Kasner SE, Chimowitz MI, Lynn MJ, Howlett-Smith H, Stern BJ, Hertzberg VS, Frankel MR, Levine SR, Chaturvedi S, Benesch CG, Sila CA, Jovin TG, Romano JG, Cloft HJ, Warfarin aspirin symptomatic intracranial disease trial investigators (2006). Predictors of ischemic stroke in the territory of a symptomatic intracranial arterial stenosis. Circulation..

[12] Bang OY (2014). Intracranial atherosclerosis: current understanding and perspectives. J Stroke.

[13] Ovbiagele B, Saver JL, Lynn MJ, Chimowitz M (2006). Impact of metabolic syndrome on prognosis of symptomatic intracranial atherostenosis. Neurology..

[14] Gouveia A, Sargento-Freitas J, Penetra J, Silva F, Machado C, Cordeiro G, Cunha L (2014). Recurrence in intracranial atherosclerotic disease: a stenosis-based analysis. J Stroke Cerebrovasc Dis.

[15] Nahab F, Cotsonis G, Lynn M, Feldmann E, Chaturvedi S, Hemphill C, Zweifler R, Johnston K, Bonovich D, Kasner S, Chimowitz M, WASID Study Group (2008). Prevalence and prognosis of coexistent asymptomatic intracranial stenosis. Stroke..

[16] Famakin BM, Chimowitz MI, Lynn MJ, Stern BJ, George MG (2009). Causes and severity of ischemic stroke in patients with symptomatic intracranial arterial stenosis. Stroke..

[17] Fu MH, Chang KC, Huang YC (2010). Recurrent ischemic stroke is predicted by intracranial large artery stenosis identified by brain MRA: an observational study of 693 patients from Kaohsiung, Taiwan. Acta Neurol Taiwan.

[18] Chang KC, Tseng MC, Weng HH, Lin YH, Liou CW, Tan TY (2002). Prediction of length of stay of first-ever ischemic stroke. Stroke..

[19] Tan T, Chang K, Liou C, Schminke U (2005). Prevalence of carotid artery stenosis in Taiwanese patients with one ischemic stroke. J Clin Ultrasound.

[20] Samuels OB, Joseph GJ, Lynn MJ, Smith HA, Chimowitz MI (2000). A standardized method for measuring intracranial arterial stenosis. Am J Neuroradiol.

[21] Chang KC, Hsu SW, Liou CW, Huang YC, Lee LH, Lui CC, Kuo YL (2010). Intra-arterial thrombolytic therapy for acute intracranial large artery occlusive disease in patients selected by magnetic resonance image. J Neurol Sci.

[22] Wu YS, Kuo YL, Wu CJ, Yip HK, Hsu SW, Liou CW, Li TH, Lin HS, Tan TY, Chen WH, Chang KC (2010). Comparison of carotid artery stenting performance between cardiologists and neuroradiologists: one medical center’s experience. Acta Neurol Taiwanica.

[23] Arenillas JF (2011). Intracranial atherosclerosis: current concepts. Stroke..

[24] Turan TN, Cotsonis G, Lynn MJ, Chaturvedi S, Chimowitz M (2007). Relationship between blood pressure and stroke recurrence in patients with intracranial arterial stenosis. Circulation..

[25] Yu DD, Pu YH, Pan YS, Zou XY, Soo Y, Leung T, Liu LP, Wang DZ, Wong KS, Wang YL, Wang YJ, Chinese intracranial atherosclerosis (CICAS) study group (2015). High blood pressure increases the risk of poor outcome at discharge and 12-month follow-up in patients with symptomatic intracranial large artery stenosis and occlusions: subgroup analysis of the CICAS study. CNS Neurosci Ther.

[26] Yahalom G, Schwartz R, Schwammenthal Y, Merzeliak O, Toashi M, Orion D, Sela BA, Tanne D (2009). Chronic kidney disease and clinical outcome in patients with acute stroke. Stroke..

[27] Said S, Hernandez GT (2014). The link between chronic kidney disease and cardiovascular disease. J Nephropathol.

[28] Holmstedt CA, Turan TN, Chimowitz MI (2013). Atherosclerotic intracranial arterial stenosis: risk factors, diagnosis, and treatment. Lancet Neurol.

[29] Orpana HM, Berthelot JM, Kaplan MS, Feeny DH, McFarland B, Ross NA (2010). BMI and mortality: results from a national longitudinal study of Canadian adults. Obesity..

[30] Toyoda K, Ninomiya T (2014). Stroke and cerebrovascular diseases in patients with chronic kidney disease. Lancet Neurol.

[31] Lee DK, Kim JS, Kwon SU, Yoo SH, Kang DW (2005). Lesion patterns and stroke mechanism in atherosclerotic middle cerebral artery disease: early diffusion-weighted imaging study. Stroke..

[32] Liebeskind DS, Cotsonis GA, Saver JL, Lynn MJ, Turan TN, Cloft HJ, Chimowitz MI, Warfarin-aspirin symptomatic intracranial disease (WASID) investigators (2011). Collaterals dramatically alter stroke risk in intracranial atherosclerosis. Ann Neurol.

[33] Fleg JL, Stone GW, Fayad ZA, Granada JF, Hatsukami TS, Kolodgie FD, Ohayon J, Pettigrew R, Sabatine MS, Tearney G, Waxman S, Domanski MJ, Srinivas PR, Narula J (2012). Detection of high-risk atherosclerotic plaque: report of the NHLBI working group on current status and future directions. JACC Cardiovasc Imaging.

[34] Leng X, Wong KS, Liebeskind DS (2014). Evaluating intracranial atherosclerosis rather than intracranial stenosis. Stroke.

[35] Alkhorayef M, Babikir E, Alrushoud A, Al-Mohammed H, Sulieman A (2017). Patient radiation biological risk in computed tomography angiography procedure. Saudi J Biol Sci.

